# The potential application of natural products in cutaneous wound healing: A review of preclinical evidence

**DOI:** 10.3389/fphar.2022.900439

**Published:** 2022-07-22

**Authors:** E Liu, Hongjin Gao, YiJia Zhao, Yaobing Pang, Yejing Yao, Zhengru Yang, Xueer Zhang, YanJin Wang, Siming Yang, Xiao Ma, Jinhao Zeng, Jing Guo

**Affiliations:** ^1^ Dermatological Department, Affiliated Hospital of Chengdu University of Traditional Chinese Medicine, Chengdu University of Traditional Chinese Medicine, Chengdu, China; ^2^ Neijiang Hospital of Traditional Chinese Medicine, Neijiang, China; ^3^ State Key Laboratory of Southwestern Chinese Medicine Resources, School of Pharmacy, Chengdu University of Traditional Chinese Medicine, Chengdu, China; ^4^ TCM Regulating Metabolic Diseases Key Laboratory of Sichuan Province, Hospital of Chengdu University of Traditional Chinese Medicine, Chengdu, China

**Keywords:** natural products, wound healing, skin, inflammation, proliferation, remodeling

## Abstract

Under normal circumstances, wound healing can be summarized as three processes. These include inflammation, proliferation, and remodeling. The vast majority of wounds heal rapidly; however, a large percentage of nonhealing wounds have still not been studied significantly. The factors affecting wound nonhealing are complex and diverse, and identifying an effective solution from nature becomes a key goal of research. This study aimed to highlight and review the mechanisms and targets of natural products (NPs) for treating nonhealing wounds. The results of relevant studies have shown that the effects of NPs are associated with PI3K-AKT, P38MAPK, fibroblast growth factor, MAPK, and ERK signaling pathways and involve tumor growth factor (TNF), vascular endothelial growth factor, TNF-α, interleukin-1β, and expression of other cytokines and proteins. The 25 NPs that contribute to wound healing were systematically summarized by an inductive collation of the six major classes of compounds, including saponins, polyphenols, flavonoids, anthraquinones, polysaccharides, and others, which will further direct the attention to the active components of NPs and provide research ideas for further development of new products for wound healing.

## Introduction

For ages, human beings relied on nature to meet their basic needs, The most critical of these are herbal medicines capable of treating a variety of diseases. Natural products(NPs) have been regarded as the active ingredients of traditional medicines and herbal medicines. The use of approximately 1,000 plant-derived substances in Mesopotamia was recorded around 2600 BC(Cragg and Newman, 2013). Natural products remain a valuable source of inspiration for the development of modern small molecule drugs. Approximately two-thirds of all small-molecule drugs approved between 1981 and 2019 are associated with NPs to some extent (Newman and Cragg, 2020). Natural products have long been a source of traditional Chinese medicine for the treatment of diseases in multiple fields, including immune and inflammatory diseases, cardiovascular and metabolic diseases, tumors, neurological diseases, as well as infectious diseases, among others (Melander et al., 2020). NPs treat not only diseases but also have great economic benefits. In 2000, 2001, and 2002, NP-derived drugs ranked among the 35 best-selling drugs worldwide (Butler, 2004). In environmental terms, as a renewable resource, natural products are able to play a greater value in the circulatory economy (Drasar and Khripach, 2019).

According to statistical data from the United States, approximately more than 6.5 million people are living with chronic wounds in the United States, and this trend is increasing. Considering the current situation, it is estimated that the medical cost may exceed $14 billion (Niu et al, 2019). The causes of wound non-healing are often because of local tissue hypoxia, repetitive trauma, and proliferation of bacteria, which, coupled with impaired cellular and host responses to stress, perpetuate injury and impede progression to the proliferative remodeling phase (Stojadinovic et al., 2008). Currently developed targeted agents are unable to meet clinical needs despite their well-defined therapeutic effects. And because the pathogenicity of multiple factors prompts current drugs in achieving wound healing less than envisioned (Zhao et al., 2016). Therefore, the development of natural products is highly necessary for addressing non-healing wounds.

Studies have shown that some NPs can act on the skin, promote wound healing, and repair the barrier because of their anti-inflammatory and antioxidant properties (Lin et al., 2017a). Moreover, the systematic induction and sorting of the modern effects of NPs of plants, animals, and algae on wound healing and their related molecular mechanisms can provide an additional therapeutic approach to nonhealing wounds.

## Pathogenesis of wound formation and healing

At the physiopathological level, wound healing is a continuous and complex process, which involves many interconnected influencing factors. (Fereydouni et al., 2019). However, several factors play an adverse role in the process of wound healing, such as malnutrition, various drugs, radiation, smoking, and hypoxia (Burns et al., 2003). Wound healing can be accelerated based on the understanding of normal tissue repair and the factors affecting the process. In addition to this, cell migration, proliferation as well as extracellular matrix (ECM) deposition is also considered important activities in wound healing. (Falanga, 2005). The normal wound healing process is a complex interplay between immune cells, signaling molecules, growth factors, and the vascular system (Ahmed and Antonsen, 2016). The normal healing of wounds includes the following eight processes: initial injury, complement, and cytokine response, neutrophil recruitment and response, macrophage recruitment and response, fibroblast activity, initiation of angiogenesis, T lymphocyte response while regulating wound closure, and proper granulation of tissue remodeling.

**FIGURE 1:**
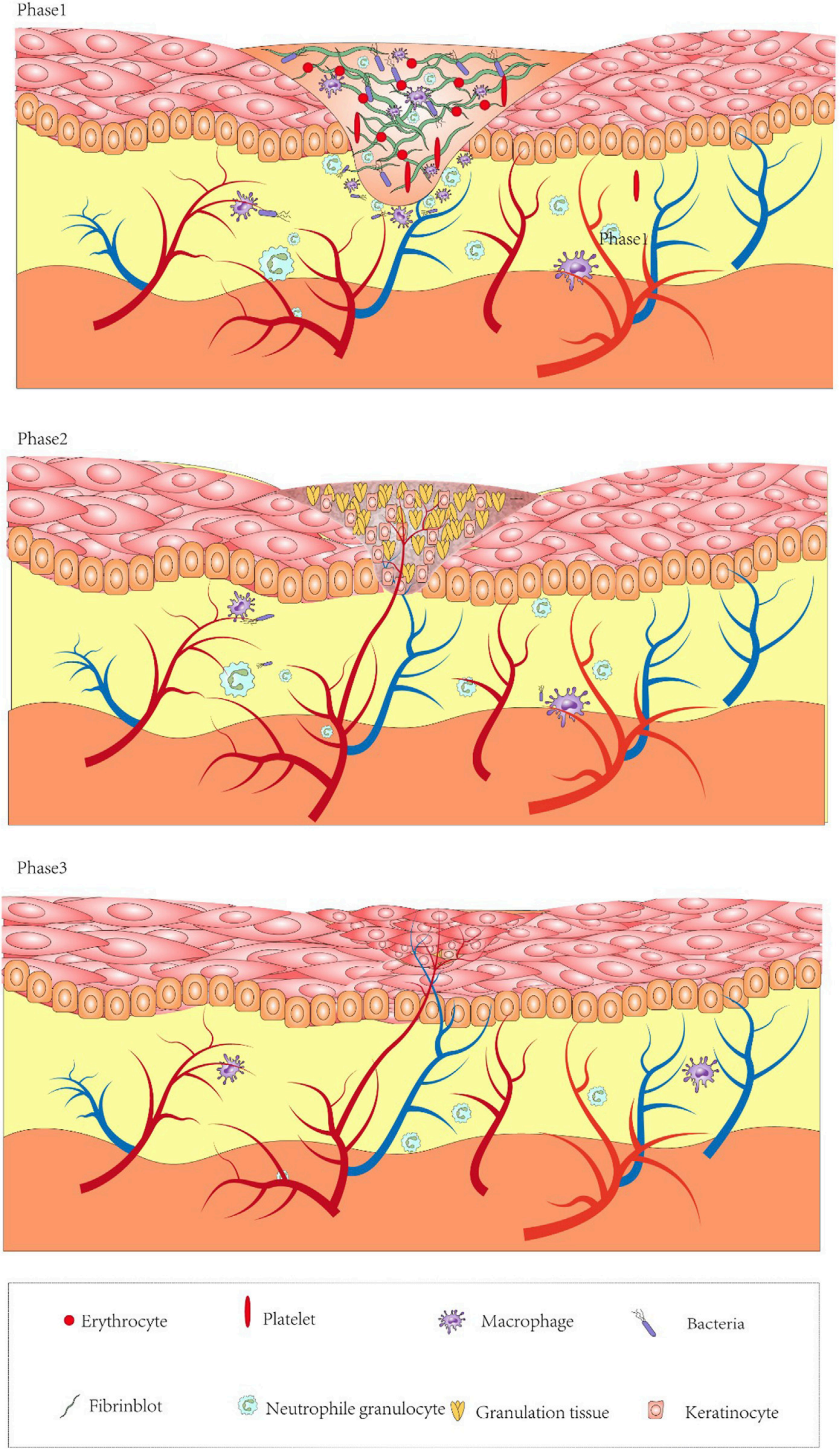
Diagram of the mechanisms of wound formation and healing. Inflammatory phase: after platelets perform initial hemostasis and release chemokines recruiting neutrophils, fibroblasts. Conversion of monocytes into macrophages engulfs necrotic tissue, decomposes bacterial products, and helps the wound to enter the proliferative phase. Proliferative phase: angiogenesis, granulation tissue regeneration, attachment of fibroblasts onto the wound. Remodeling phase: fibroblasts and myofibroblasts continuously differentiate to form tight cross links.

Wound healing mainly includes the following three stages (Figure 1): “Inflammation, proliferation, and remodeling” (Han and Ceilley, 2017). Although these stages are separated for simplicity, they overlap several times, and even different wound areas may be in different healing stages. Any interruption in the healing process will disrupt the subsequent stage and may lead to wound collapse for a long time (Gantwerker and Hom, 2011).

Coagulation is a necessary process to stop bleeding and protect wounds (Baron et al., 2020). Bleeding from the wound exposes platelets to the subendothelial layer of thrombosis. Platelets provide initial hemostasis and also release various cytokines (insulin-like growth factor-1, tumor growth factor [TGF] -α, TGF-β, and platelet-derived growth factor [PDGF]), and hormones and chemokines allow macrophages and fibroblasts to enter the wound (Sorg et al., 2017). Even more, at 5–10 min of injury, the injured vessels undergo vasoconstriction to reduce blood loss, and the tissue gap is filled with thrombus. They are composed of cytokines and growth factors (Buganza-Tepole and Kuhl, 2013). At this time, fibronectin can be found in the blood clot. Coagulation and platelet degranulation activate the inflammatory phase, and the chemokines released by these platelets are important in the recruitment of leukocytes (mainly neutrophils) and stem cells or fibroblasts in wounds (Pazyar et al., 2014). Neutrophils produce elastase, collagenase, TNF-α, and interleukin-1 (IL-1), which will recruit fibroblasts and epithelial cells (Lee et al., 2012). Monocytes transform into macrophages, engulf necrotic tissue, disintegrate neutrophil fragments and bacterial products, and release platelet-derived growth factor (PDGF) and TGF-β. β-Fibroblast growth factor (FGF), TNF-α, IL-1, and IL-6 help heal the proliferative stage, promote collagen synthesis, secrete epidermal growth factor (EGF), promote vascular endothelial cell proliferation and angiogenesis, and play a crucial role in the proliferation of wound tissue (Khanna et al., 2002; Buganza-Tepole and Kuhl, 2013; Martin and Nunan, 2015).

Four to five days after injury is approximately the time at which the proliferative phase appears, even lasting several weeks. It includes angiogenesis, granulation tissue, and ECM formation and re-epithelialization (Yip, 2015). At the same time, the tissue recruits fibroblasts from the surrounding intact tissue. Endothelial cells undergo migration under the stimulation of vascular endothelial growth factor (VEGF) to promote the formation of new blood vessels (Yun et al., 2010). The newly formed capillaries are connected with adjacent capillaries migrating in the same direction and forming granulation tissue. Fibroblasts are attracted to the wound and produce ECM and contain high levels of immature type III collagen (col3), which is completely different from type I collagen (col1) seen in normal skin and mature scars (Zielins et al., 2015). The highest concentration of collagen in the wound is found approximately 3 weeks after the initial injury. Moreover, 3–5 days after injury, fibrin embolus is replaced by granulation tissue, and the wound begins to shrink. At this stage, growth factors and other peptides are crucial in the supplement and direction of cells. VEGF, (PDGF and FGF protein family (particularly FGF-2) are all associated with angiogenesis and tissue repair (Yun et al., 2010; Hu et al., 2014). Finally, it enters the last stage of wound healing, which is called the remodeling stage.

Reconstruction is a dynamic process, starting from the end of granulation tissue development (Landén et al., 2016). A series of key events in the process of wound healing and tissue repair is the effective differentiation of fibroblasts and myofibroblasts, including mechanical tension and cytokines (TGF-β, PDGF, and FGF). Drive fibroblasts to differentiate into myofibroblasts, and myofibroblasts express α-smooth muscle actin, allowing the wound to form a close cross-linking and increasing the tensile strength of mature scars up to 80% of uninjured skin, In addition, with the transformation of immature col3 into mature col1, the ratio of skin stretching col3 and col1 decreases (Sgonc and Gruber, 2013; Landén et al., 2016; Opneja et al., 2019), and this process can last up to 2 years.

## NPs treatment for wound healing

**FIGURE 2:**
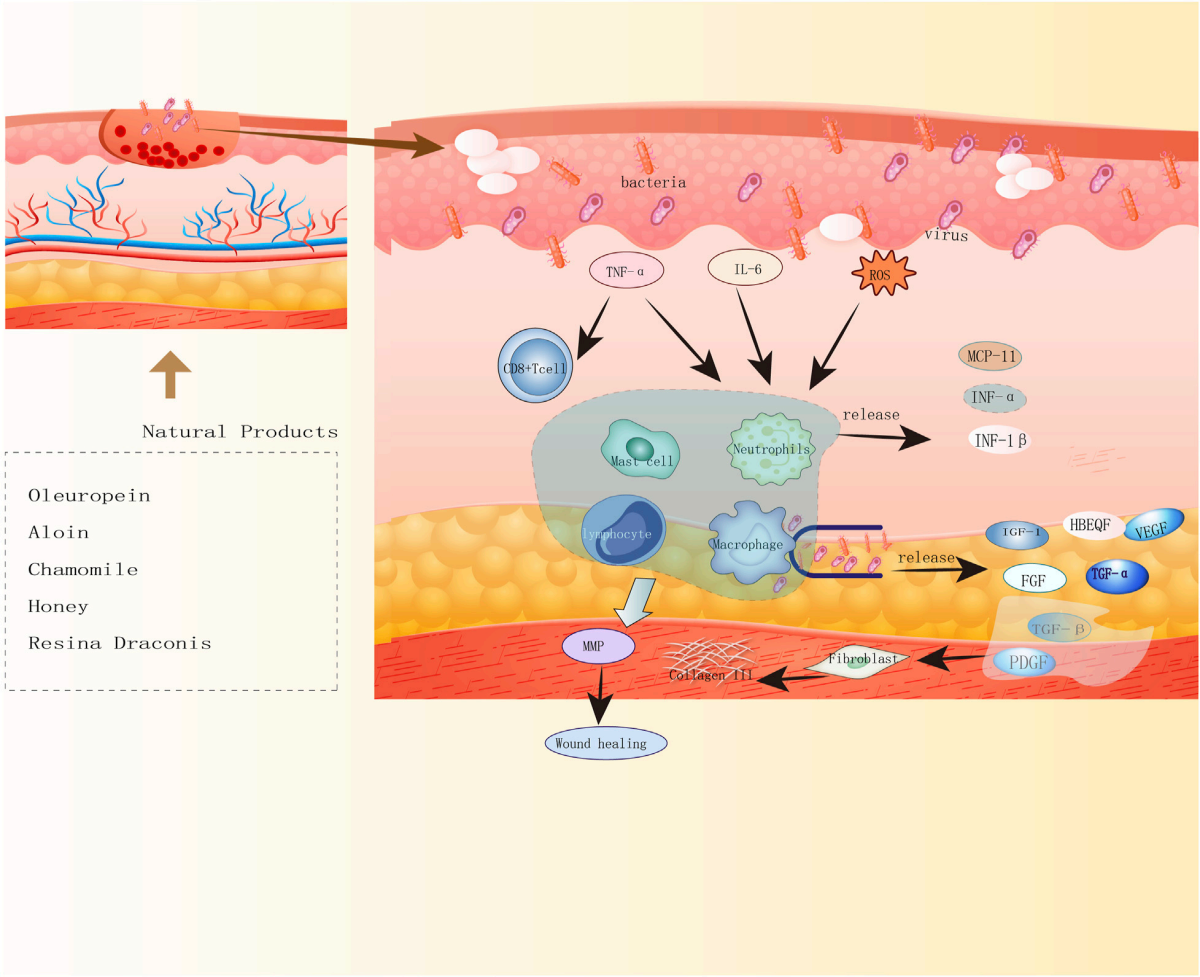
Diagram of natural product antimicrobial mechanisms. Five natural products of oleuropein, aloin, chamomile, honey, Resina Draconis promote wound healing by antibacterial, antiviral mechanisms.

**FIGURE 3:**
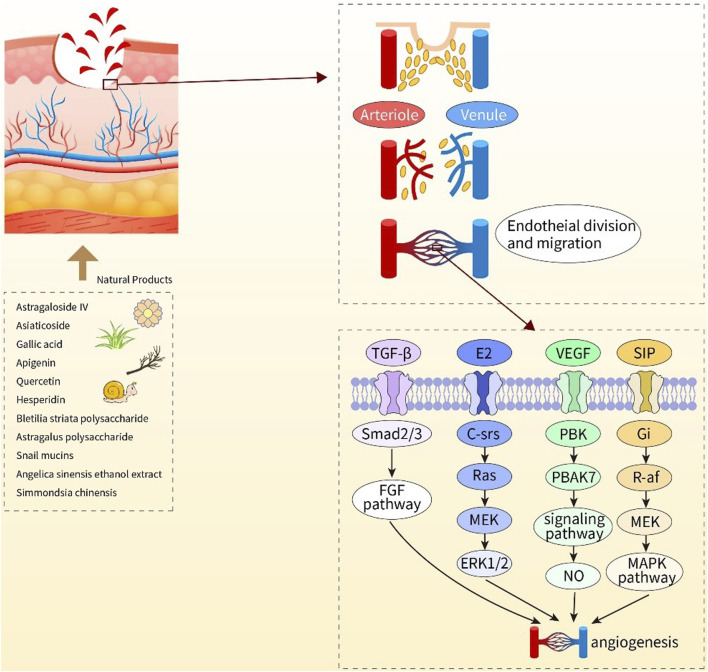
Diagram of the mechanisms of action of natural products to promote angiogenesis. Astragaloside IV, asiaticoside, gallic acid, apigenin, quercetin, hesperdin, bleilia striata polysaccharide, astragalus polysaccharide, snail mucins, angular sinensis ethanol extract, Simmondsia chinensis, the above 11 natural products play a positive role in wound healing by promoting angiogenesis.

**FIGURE 4:**
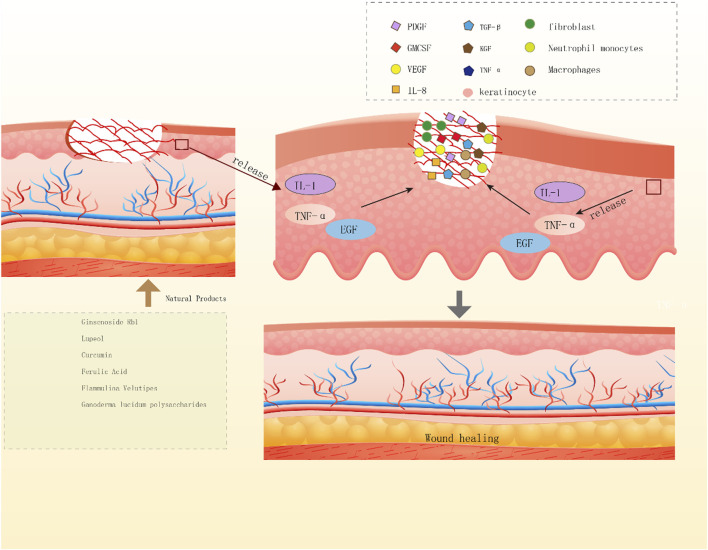
Diagram of the mechanisms by which natural products promote wound re epithelialization. Ginscnoside Rb1, lupeol, curcumin, ferulic acid, Flammulina velutipes, Ganoderma lucidum polysaccharides, the above six natural products release IL-1, TNF - α and EGF by keratinocytes to promotes wound re epithelialization.

NPs have diverse biological activities (Melander et al., 2020), and a large proportion of newly developed drugs are produced from NPs (secondary metabolites) and compounds derived from them (Li and Vederas, 2009). Like saponins, polyphenols, flavonoids, anthraquinones, and polysaccharides, NPs play crucial roles in wound healing. Saponins can promote vascular regeneration and shorten wound healing time. Moreover, studies have revealed that polyphenols, widely present in the human diet, can improve wound healing because of their beneficial factors such as antioxidant, immunomodulatory, and bacteriostatic activities (Paszkiewicz et al., 2012). Flavonoids occur in a wide range in nature, such as food, vegetables, and fruits, and their compound inhibitors can kill several bacterial strains and attenuate the inflammatory process, which is beneficial for wound healing (Havsteen, 2002; Silva et al., 2021). Anthraquinones, which have hemostatic antibacterial effects, and their beneficial components will contribute to the hemostatic phase of wound healing and the inflammatory phase (Masi and Evidente, 2020). Polysaccharides are essential components of higher plants, animal cell membranes, and microbial cell walls (Yu et al., 2018). At present, polysaccharide-based scaffolds have been developed and used in wound healing, and they have high moisturizing ability and are nontoxic, thus ideal for humans (Nosrati et al., 2021). The newly developed hydrogels of polysaccharides can provide suitable moisture to wounds and also act as a barrier against bacteria. Although the effectiveness of NPs varies, they play a role in the different phases of wound healing (Figures 2, 3, 4).

## Saponins

### Ginsenoside Rb1

**FIGURE 5:**
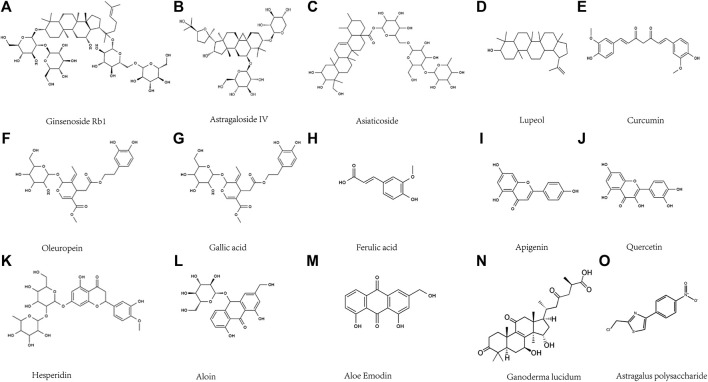
Diagram of the chemical formula structure of natural products.

Ginsenoside Rb1 (Figure5A) belongs to one class of saponin compounds. Saponins are a diverse group of compounds widely distributed in the plant kingdom (Güçlü-Ustündağ and Mazza, 2007). As a traditional medicine, ginseng is known as the king of herbal medicine. It has been used as a treatment for diseases in Asian countries for thousands of years (Ratan et al., 2021). Ginsenoside Rb1 is the main active component of ginseng and belongs to sterols (Lee et al., 2018). Ginsenoside Rb1 can accelerate wound epithelialization and intervene to accelerate PDGF-BB and PDGF receptor-β and the peak expression of FGF-2 protein and mRNA, promote wound healing and reduce wound healing time. It can be inferred that ginsenoside Rb1 can regulate PDGF-BB/PDGF receptor-beta signal pathway to regulate the expression of FGF-2to have a therapeutic effect on wound healing (Zhang et al., 2021). *In a vivo and vitro model,* ginsenoside Rb1 was able to promote neovascularization around the wound and increased VEGF, IL-1β in the regenerated skin area and expression by macrophages. Besides, Ginsenoside Rb1 at concentrations from 100 fg ml^−1^ to 1 ng ml^−1^ enhanced the VEGF production and HIF-1α expression induced by IL-1β in HaCaT cells. (Kimura et al., 2006). Ancient Chinese have always spoken ginseng as a supplement. And subsequent studies have found that several ginsenosides isolated from P. ginseng have beneficial effects on multisystem diseases in the body, which is enough to illustrate the safety of ginsenosides (Ahmed et al., 2016).

### Astragaloside IV

Astragaloside IV (AS-IV, Figure 5B) is an organic substance and a drug extracted from *Astragalus membranaceus*. To date, many cellular and animal studies have shown that the neuroprotective, hepatoprotective, anticancer, as well as antidiabetic effects of as-iv, can be attributed to its antioxidant, anti-inflammatory as well as anti-apoptotic properties (Zhang et al., 2020). AS-IV could stimulate wound healing, improve the strength of repairing skin, promote angiogenesis and collagen synthesis, and reduce collagen I/III and TGF-β 1 secreted by fibroblasts level. *In vivo* studies showed that (Chen et al., 2012) AS-IV was able to stimulate the wound to recover faster after topical injection in a rat model of full-thickness cutaneous excisional wounds. Angiogenesis occurred at the wound site after 30 days of treatment. Wang et al. demonstrated that as-iv could increase the protein expression in HUVECs by activating the sumo pathway, which would further explain how as-iv promotes the proliferation and migration of vascular endothelial cells, antagonizes the poor microenvironment of hypoxia and high glucose, and improves angiogenesis and wound healing *in vitro* and *in vivo* (Shen et al., 2021). In addition, AS-IV hydrogel is effective in repairing skin wounds and significantly Promotes wound healing, increases collagen synthesis as well as restores skin tensile strength. At the same time, AS-IV hydrogel activates skin appendages and increases TGF-β1 in serum (Peng et al., 2012). AS-IV loaded with solid lipid nanoparticles-enriched hydrogel gel proved to be excellent local preparation for wound healing and scar resistance (Chen et al., 2013). Interestingly, AS-IV not only has the effect of Astragalus polysaccharide, but its drug efficacy is also more than 30 times that of Astragalus polysaccharide, so it can be used as a skin-healing agent.

### Asiaticoside

Asiaticoside (AS, Figure 5C) is extracted from the medicinal plant *Centella asiatica*. It grows in Asia, where it is abundant in India, Pakistan, as well as Madagascar (Brinkhaus et al., 2000). In skin diseases, *Centella Asiatica* can improve the healing of small wounds, scratches, burns, and hypertrophic wounds, and exert anti-inflammatory and antibacterial efficacy (Gohil et al., 2010; Antognoni et al., 2011). Maquart and colleagues believe that asiaticoside can promote wound healing through fibroblast proliferation, and this mechanism may be associated with the migration of tissue cells around the wound or the expression and activation of certain growth factors (Maquart et al., 1999). Asiaticoside induces the synthesis of col1 in human dermal fibroblasts through the phosphorylation of Smad 2 and Smad 3 and further binding Smad 3 and Smad 4 (Lee et al., 2006). *In vivo* and *in vitro* studies showed that asiaticoside exhibited remarkable healing ability in both the normally healed wound model and the long-standing unhealed wound model (Shukla et al., 1999a). In excised rat skin wounds, The enzymatic and nonenzymatic antioxidants were able to be increased by topical application of 0.2 concentration of Asiaticoside twice a day for 7 days. (Shukla et al., 1999b).

### Lupeol

Lupeol (Figure 5D) is a pentacyclic triterpenoid saponin, present in the epidermis of lupeol seeds and the latex of fig trees and rubber plants, and in strawberries, mangoes, grapes, olives, and other fruits. It can be used to treat various types of diseases such as arthritis, diabetes, as well as skin wounds (Barros et al., 2010). Lupeol participates in the closure of skin wounds by stimulating the migration of keratinocytes and the contraction of fibroblasts embedded in a collagen matrix (Wardecki et al., 2016). Studies have shown more significant wound healing activity with topical lupeol ointment than control, increased granulation tissue, and more efficient wound healing in a dead space wound model (Harish et al., 2008). *In vitro* studies showed that to reduce keratinocyte and fibroblast proliferation, the dose of lupeol was about 20 µg/ml. Lupeol may activate PI3K/Akt and p38 MAPK, NF- κ B signaling, inhibition of keratins as well as MMP-2 and Tie-2 to promote wound healing. (Pereira Beserra et al., 2018). In terms of experimental indicators, *in vivo*, experimental studies show that in the lupeol treatment group, the expression of NF-κβ and IL-6 decreased significantly and the level of IL-10 increased. Lupeol also increased FGF-2, TGF-β1, HIF-1α, and the expression of HO-1 and superoxide dismutase (SOD)-2 (Beserra et al., 2019). Besides that, on one hand, lupeol cream was able to modulate NF- κ B expression to exert anti-inflammatory effects and promote granulation tissue growth, vascular regeneration as well as re epithelialization. On the other hand, it can regulate Ki-67, VEGF, EGF, and TGF- β 1 as well as stimulating the synthesis of collagen fibers to promote tissue remodeling (Pereira Beserra et al., 2020).

## Polyphenolic compounds

### Curcumin

Curcumin (Figure 5E) is a polyphenol compound. As a yellow dye, the main active ingredient of curcumin widely exists in the medicinal plant turmeric (Trigo-Gutierrez et al., 2021). Modern research shows that curcumin has a wide range of pharmacological effects, including anti-inflammatory antioxidation, and antibacterial effects (Witika et al., 2021). Curcumin may play a crucial role in wound healing due to its antioxidant and anti-inflammatory functions. Four processes such as the formation of granulation tissue, collagen deposition, tissue remodeling as well as wound contraction may all receive the influence of curcumin, thereby promoting the healing of wounds. (Zhao et al., 2019). One study found that (Rujirachotiwat and Suttamanatwong, 2021) found that curcumin significantly upregulated the mRNA expression of col1, keratinocyte growth factor-1, and epidermal growth factor receptor (EGFR) in human gingival fibroblasts *in vitro* wound healing model. ERK signaling pathway is essential for curcumin-induced col1 and EGFR mRNA expression. Gong and colleagues (Gong et al., 2013) tested a dressing made of curcumin thermosensitive gel *in vitro* experiments. Compared with other groups, animal wound healing with curcumin heat-sensitive dressing was significant, with a higher degree of re-epithelialization, well-arranged granulation tissue, and fibroblast deposition. In addition, as a skin wound healing agent, curcumin has been identified to produce TGFβ1 in the promotion of wound healing. TGFβ1 can stimulate fibroblasts of the tissue surrounding the wound to proliferate and express appropriate integrin receptors, thereby moving to the wound site to favor wound healing (Hong et al., 2008; Thi et al., 2020). Interestingly, the fad also identified curcumin as a safe compound. Clinical data studies revealed that topical curcumin showed good bioactivity, which will provide a useful aid for the further development of effective composite formulations (Vollono et al., 2019).

### Oleuropein

Oleuropein (OLE, Figure 5F) is a phenolic compound found in the olive plant. It has crucial anti-inflammatory and antioxidant effects. Some studies proved that ole exerted anti-inflammatory activity may be related to the production of lipoxygenase activity, leukotriene B4 (Motawea et al., 2020). In addition, the biosynthesis of pro-inflammatory cytokines or inflammatory parameters is also associated with its inhibitory effects (de la Puerta et al., 1999). Results indicated that ole could exert obvious favorable effects in the cutaneous wound healing of aged male Balb/c mice (Mehraein et al., 2014). *In vivo study* showed that the wound closure time was shortened when the diabetic rats and non-diabetic rats were treated with ole wound dressing topically. The above findings indicate that ole wound dressing is a suitable choice for the treatment of diabetic wounds (Samancio et al., 2016). The acute toxicity test results found that the mice did not experience any side effects or fatal situations even when the dose reached 1,000 mg/kg (Zheng et al., 2021).

### Gallic acid

Gallic acid (GA, Figure5G) is a polyphenolic organic compound widely found in plants such as Rheum palmatum, EucalyptusGrandiss, and Cornus. GA has antioxidant, anti-inflammatory, and analgesic effects (Comino-Sanz et al., 2021). Studies have shown that GA can improve wound healing in diabetic rats, and promote neovascularization, and collagen deposition during wound healing in diabetic animals (Singh et al., 2019). *In vitro studies* have shown that GA upregulates the expression of antioxidant genes, stimulates cell migration on fibroblasts, and activates healing factors (Yang et al., 2016). More notably, GA can not only be extracted from many plants and can be produced in large quantities by biological and chemical synthesis. Taking a step further, GA hardly has any obvious toxicity and toxic side effects in any animal experiments and clinical experiments (Bai et al., 2021).

### Ferulic acid

Ferulic acid (Figure 5H) is a polyphenol and the most common compound in whole grains, spinach, parsley, grapes, rhubarb, and cereal seeds. It has low toxicity and several physiological functions. Such as exerting anti-inflammatory, antimicrobial, antioxidant, anticancer as well as anti-diabetic effects. (Zduńska et al., 2018). *In vivo studies* showed that ferulic acid accelerated wound regeneration and healing. The granuloma attack and epithelization of the ferulic acid group were faster than the control group (Dwivedi et al., 2022). The use of ferulic acid ointment during healing can inhibit lipid peroxidation and increase catalase, superoxide dismutase, and glutathione (Ghaisas et al., 2014). Considering the safety of drugs, ferulic acid, as an important potential therapeutic agent, has the characteristics of low cost, easy availability, low toxicity, and few side effects (Kaur et al., 2022).

## Flavonoids

### Apigenin

Apigenin (Figure 5I) is a common flavonoid compound that widely exists in fruits and vegetables (such as onion, orange, and parsley) (H u et al., 2021). Overall, the exertion of its pharmacological effects is mainly related to anti-inflammation, antioxidation, andante-cancer. Studies have shown that topical application of apigenin gel resulted in faster wound re-epithelialization and improved inflammation (Lopez‐Jornet et al., 2012). The findings revealed that apigenin combined with LysGH15 acts as a topical antibacterial agent against *Staphylococcus aureus* (Cheng et al., 2018). In plastic surgery, the integrity of wound healing is extremely crucial. Apigenin can promote angiogenesis in mice, promote the survival rate of the skin flap area, and reduce tissue edema (Zhu et al., 2021). Up to now, apigenin is considered safe and, even at high doses, there have been no reports of toxicity. However, at high doses, it is able to elicit muscle relaxation as well as produce sedation (Ross and Kasum, 2002).

### Quercetin

Quercetin (Figure 5J) belongs to flavonoids, which are widely distributed in the stems, leaves, flowers, skins, seeds, and fruits of plants, such as apples, grapes, onions, tea, tomatoes, and Ginkgo biloba (Li et al., 2016). Quercetin mainly exerts anti-inflammatory and antioxidant activities and improves immunity. *In vitro* experiments, the mechanism by which quercetin exerts anti-inflammation activity is associated with the inhibition of the cyclooxygenase (COX)and lipoxygenase (LOX) (Kim et al., 1998); Moreover, quercetin can induce inflammation by increasing peroxisome proliferator-activated receptor C (PPAR-γ) In activity to indirectly prevent inflammation and thereby antagonize NF-κB or transcriptional activation of activator protein-1 inflammatory genes (Li et al., 2016). Studies have shown that after quercetin binds to collagen, the hydroxyproline concentration in granulation tissue has increased from 0.78 mg/ML in controls to 1.84 mg/ML, indicating enhanced collagen production in granulation tissue (Hatahet et al., 2016). Cytokines, growth factors, and proteases play critical roles in wound healing. The findings suggest that quercetin ointment at a topical 0.3% w/w concentration can promote better wound outcomes in a rat cutaneous diabetic wound model, which may be related to the reasons mentioned above (Kant et al., 2021). As quercetin is poorly water-soluble, improving the permeation ability to increase the stability is the main purpose of quercetin nano dosage forms (Brüll et al., 2015). therefore, the current nanoformulation with quercetin as the main raw material comes with an age limit.

### Hesperidin

Hesperidin (Figure 5K) is a flavonoid found in the peel of citrus fruits and is an antioxidant found in plants, that is, essential for human health. It has recently been shown to exert several functions on the skin (Man et al., 2019), including wound healing, UV protective, anti-inflammatory, antimicrobial, antioxidant, and whitening effects. The study revealed a significant increase in the wound closure process at 24 h after the addition of hesperidin containing 0.05% to the culture medium. and administration of hesperidin significantly upregulated TGF-β in wound tissues and Smad-2/3 mRNA expression (Li et al., 2018). In addition, topical or oral hesperidin can shorten the wound healing time of irradiated mice, which was 3 days,s and may be associated with the inhibition of NF-кB. COX-II and LOX have been associated with their transcriptional activation (Jagetia and Rao, 2022). Moreover, The results demonstrated that topical hesperidin enhanced epidermal skin permeability and barrier homeostasis in mice and further stimulated epidermal proliferation and differentiation (Hou et al., 2012). In general, hesperidin appears to be very safe with no adverse effects even during pregnancy. In animal experiments (Garg et al., 2001), phosphorylated hesperidin is nontoxic to organisms and tissues, readily absorbed, and does not cause allergic reactions.

## Anthraquinone compounds

### 1Aloin

Aloe Vera (Figure 5L) is one of the major active components of the plant aloe vera, belonging to anthraquinone compounds. Aloe protects the skin mainly through anti-inflammatory and antioxidant mechanisms (Sánchez et al., 2020). A study suggested that the mechanism by which aloe glycoside exerts partial anti-inflammatory activity may be related to the inhibition of the reactive oxygen species (ROS) - mediated JAK1-STAT1/3 signaling pathway in RAW264 macrophages (Ma et al., 2018). Moreover, aloe glycoside was able to produce skin protective effects. This may be related to decreased IL-8, DNA damage, lipid peroxidation, and reactive oxygen species production (Liu et al., 2015). In mouse skin samples, macrophages and neutrophils were increased at the site of injury after Aloesin treatment, which illustrates that aloesin exerts a positive effect on inflammation at an early stage. In addition, aloesin can promote neoangiogenesis, which may be related to an increase in TGF- β1 is associated (Wahedi et al., 2017). In recent years, one of the reasons for the increasing number of aloe external preparations may be that it plays a role in promoting skin healing.

### Aloe-Emodin

Aloe-emodin (Figure 5M) is an anthraquinone compound in aloe. It can be used in the development of pharmaceutical raw materials, intermediates, and anticancer drugs. It has broad application prospects in the field of medicine. *In vitro* studies show that aloe-emodin can protect against burn wounds (Lin et al., 2017b). The activity of forex-isolated compounds (aloe-emodin, chrysophanol, and aloin) was investigated by Kambiz et al. (Kambizi et al., 2005). They found that aloe-emodin and along A exhibit inhibitory activities against a variety of bacteria a. Existing evidence suggests that the use of multiple dosages of aloe may effectively enhance the wound healing process compared with traditional treatment methods (Maenthaisong et al., 2007). This study confirmed that increased wound healing occurs following treatment with aloe-emodin, thus supporting the use of aloe vera plants to improve burn wound healing (Lin et al., 2016). However, adverse effects of aloe-emodin have been reported, including phototoxicity, hepatotoxicity, and nephrotoxicity (Dong et al., 2020). Therefore, we should look at this problem dialectically.

## Polysaccharides

### Bletilla striata polysaccharide

*Bletilla striata* polysaccharide (BSP) is typically considered the main active component of *Bletilla striata*. It is an orchid, mainly distributed in southern China. More and more shreds of evidence show that BSP has various activities, including immune regulation, anti-inflammatory, wound healing, and antioxidant. Studies have shown that Bletilla polysaccharides can promote the growth of endothelial cells and enhance the self-secretion of endothelial growth factors r (Wang et al., 2006). The root whiskers of Bletilla striata are extremely valuable and able to unite with pseudoscalar, both of which share similar chemical compositions (Jiang et al., 2013). BSP could promote endothelial cell proliferation and VEGF expression and enhance NO synthase, TNF- α, And IL-1 β Levels of mRNA (Diao et al., 2008; Hung and Wu, 2016). BSP shows good healing function and can regulate macrophages during inflammation and proliferation, which will further exert its good potential to promote wound healing. (Chen et al., 2018). In the *in vivo* study, BSP hydrogel was applied on the mouse wound skin with a good wound healing effect, infiltrating inflammatory cells and TNF- α attenuated, But the secretion level of EGF increased (Luo et al., 2010). At present, BSP-based derivatives are also constantly being developed, which paves the way for promoting wound healing.

### Flammulina velutipes

In recent years, edible bacteria have become a type of food with a high nutritional value because of the umami taste and rich nutrition. Flammulina Velutipes (FVP) is one of the most common economic food bacteria (Yuan et al., 2019). FVP polysaccharide is one of the active components derived from FVP. TG05 is a major monomer composed of extracted components of a wide range of sugars. Such as xylose, and glucose. It is the residue of the culture process of FVP. But also has the ability to induce the proliferation and migration of human keratinocytes. This depends mainly on the timing of the experimental design and the doses used (Xu et al., 2019). Interestingly, the novel seeded scaffold, prepared from FVP by cryosection “processing”, can promote the regeneration of hair follicles and injured tissues (Chen et al., 2021). This novel technique will further facilitate the study of nonhealing wounds.

### Ganoderma lucidum polysaccharides

Ganoderma lucidum Polysaccharide (GL-PS; Figure 5N) is a substance extracted from the spore meal or Ganoderma lucidum and is one of the most effective components of Ganoderma lucidum. Research has found that it could help in avoiding the delayed wound healing and improve wound angiogenesis in STZ-induced type 1 diabetic mice models by inhibiting cutaneous MnSOD nitration, p66Shc, and mitochondrial oxidative stress (Tie et al., 2012). *In vitro studies* revealed that 10, 20, and 40 µg/mL GL-PS significantly increased the viability, promoted the migration, and upregulated the cell-in-cell phenomenon and TGF-β in fibroblasts. The expression of Wnt/β, which may be related to the activation of Wnt/β and TGF-β1, was associated with the upregulation (Hu et al., 2019). Previous studies have shown that Wnt/β-catenin signaling may be involved in wound healing (Liu et al., 2012), and GL-PS may also be implicated in this signaling pathway for wound healing. Notably, in vitro studies, GL extracts exhibit toxic effects when present at higher concentrations in cells than required for a stimulatory effect (Bishop et al., 2015).

### Astragalus polysaccharide

Astragalus Polysaccharides (APS, Figure 5O) can be produced from the main active components extracted from the traditional Chinese medicine Astragalus membranaceus. Modern pharmacological studies believe that most of the active components that Astragalus plays a role in the process of wound healing are polysaccharides and saponins. *In vitro experiments*, aps2-1 purified from Astragalus membranaceus can cause IκBα and cyclin D1 to reduce mRNA and protein expression levels, This may be related to wound healing (Zhao et al., 2017). *In vivo study*, Astragalus polysaccharide-loaded fibrous mats promoted the recovery of microcirculation in and around the skin wound, and mouth, which may be associated in a dose-dependent manner to promote blood flow in the surrounding skin and increase the endocrine and microvessel density in the regenerated skin tissue (Yang et al., 2015). In addition, Astragalus gum extracted from Astragalus polysaccharide for back wounds in mice has beneficial effects in accelerating wound reduction as well as skin healing (Fayazzadeh et al., 2014). Interestingly, Astragalus extracts (APS and astragaloside saponins) are safe without significant toxicity and side effects. Its safe dose is 5.7–39.9 g/kg in rats and 2.85–19.95 g/kg in dogs (Yu et al., 2007). Therefore, there is a certain basis for applying Astragalus polysaccharides to wound healing.

## Others

### Honey extract

Honey is available as both a food and a natural product. It contains various phenolic compounds, enzymes, and sugars and is known for its antioxidant, anticancer, anti-inflammatory, and antibacterial properties. (Scepankova et al., 2021). Honey has been used in wound dressings for thousands of years. A survey showed that compared with nonantibacterial drugs, the average time of wound healing after intervention with honey was reduced by 5.32 days (Norman et al., 2017). The findings suggest that honey can reduce the level of inflammation, edema, and pain by reducing the activities of COX1 and COX2 (Nooh and Nour-Eldien, 2016; Yadav et al., 2018). *In vitro*, honey can promote angiogenesis, which may be associated with peroxidation, and induce macrophages to release VEGF for stimulating angiogenesis (Rossiter et al., 2010). In recent years, honey products have had great potential in wound healing. Whether directly applied or mixed with fiber or hydrogel membrane, it has achieved a good curative effect (Molan and Rhodes, 2015; Sen et al., 2021). It is worth noting that the poisoning symptoms caused by honey may differ depending on the source of the toxin (Islam et al., 2014). Honey should not be considered a completely safe food.

### Comfrey extract

Comfrey is a perennial herb in the genus *Shikonin* of the family purpura, belonging to the family Boraginaceae (Staiger, 2012), and extracts from its leaves and roots have been used in the treatment of wounds. In a clinical trial, the wound healing time was significantly shorter after the topical application of an ointment of a comfrey extract preparation (Staiger, 2012). In addition, in children, a 10% concentration of *Shikonin* extract formulation was applied topically to wounds, and its healing rate was higher than that of a 1% concentration (Barna et al., 2012). In addition to that, some extracts with comfrey as raw materials are widely used in clinical practice, such as comfrey cream, comfrey oil, etc. Unfortunately, Comfrey is recognized as hepatotoxic as well as has carcinogenic potential for human health (Mei et al., 2010).

### Chamomile extract

Chamomile is one of the flavonoids, mainly from the flowers and leaves of the plant chamomile (Hussain et al., 2017). Studies have shown that chamomile extract has antioxidant (Singh et al., 2011) effects. *In vitro studies* have shown that chamomile extract can produce anti-inflammatory effects by interfering with the COX-2 pathway (Srivastava et al., 2009). In the *in vivo* study, chamomile extract was applied to a rat skin burn model, applied twice a day, and after 61 days a clear wound healing was visible (Jarrahi, 2008). Some findings showed that topical application of 0.04 ml/day of chamomile ointment treated wounds on the rat tongue over 10 days, re-epithelialization of the wound as well as collagen fiber formation (Duarte et al., 2011). The results of this study show that tribo oil from chamomile extract is able to additively accelerate wound healing in rats. As technology continues to update, materials such as hydrogels are also able to be combined with chamomile extracts, These results suggest that starch/extract/4 wt% nZ dressing significantly promoted wound healing (Salehi et al., 2017).

### Resina draconis

Resina Draconis (RD) is a red resin, which extracts from the trunk of calcaneal cochinensis, which mainly grows in Yunnan and Guangxi provinces (Vaníčková et al., 2020). It has antithrombotic, antioxidant, antiseptic, and other effects (Choy et al., 2008). In the animal model, after using Resina Draconis extract and burn ointment, the time of wound re-epithelization was significantly shortened, which could restore the integrity and protect from infection (Liu et al., 2013). The expression of TGF-β1 and VEGF increased significantly in wound tissue of the skin, which are the main genes for wound healing (Santos et al., 2007). In clinical studies, the use of Longxue cream can significantly improve the wound healing time (Namjoyan et al., 2015), which may be because of the formation of a protective layer of polyphenols on the surface of the wound, and acts as a physical barrier, thus shortening the inflammatory process (Chen et al., 1994).

### Simmondsia chinensis

Simmondsia Chinensis is a plant with a tenacious life span and drought tolerance ability. The oil produced from it is the main biological source of wax fat, which can be used for various skin injuries, including skin infections and wounds (Pazyar et al., 2013). *In vitro*, jojoba liquid wax significantly enhances the wound closure of keratinocytes and fibroblasts. Its mechanism of action is strictly dependent on the flow of calcium ions. Besides that, the involvement of multiple signal pathways including PI3K Akt mTOR, p38, and ERK1/2 MAPK was involved (Ranzato et al., 2011). In addition, jojoba is often used as a food additive, showing high safety and no significant toxicity and side effects (Ibrahim et al., 2011).

### Snail mucins

Recently, snail mucins have also been viewed as a profitable resource and used as skin care products, wound healing agents, surgical gels, and anti-gastric ulcer agents (McDermott et al., 2021). In addition, studies have shown mucus secretions to be more resistant to infection than some antibiotics (e.g., amoxicillin and streptomycin) (Gugu et al., 2020). Snail mucus can promote wound healing and has emerged as a base substance for various biomaterials (Park, 2011). In mouse models, snail secretory filtrate (SSF) significantly increased the rate and percentage of wound area closure and COL3A1 level and decreased MPO expression in the wound and IL-1β. Moreover, TNF-α expression levels of the restorative growth factor TGF-β in wounds level up, suggesting an enhancement of the adaptive wound healing process (Gugliandolo et al., 2021). This illustrates that snail extract is a safe and effective alternative therapy for wound healing (Tsoutsos et al., 2009).

### Angelica sinensis ethanol extract

The ethanol extract of Angelica Sinensis comes from Angelica Sinensis. Studies have shown that (Zhang et al., 2017) both *in vivo and in vitro studie*s, *Angelica Sinensis* ethanol extract (ASEE) can produce obvious effects on angiogenesis. It has been shown to improve wound healing in diabetic rats *in vivo* studies, a mechanism that may also derive from effects exerted by angiogenesis. *In vitro study* found that (Bai et al., 2012a) the concentration was 5 × 10^(−4)^∼5 × 10^(−2)^ g/L of ASEE can significantly promote the proliferation of human normal fibroblasts, accelerate the cell cycle of fibroblasts, upregulate the expression of collagen I and collagen III, and enhance wound healing. Interestingly, ASEE could accelerate the cell cycle of KC, and inhibit cell apoptosis. This may be associated with the downregulation of cyclin D1 mRNA and caspase-3 mRNA. Such findings suggested that the pathway by which ASEE promoted wound healing was associated with accelerated wound re-epithelialization. (Bai et al., 2012b).

## Conclusions and perspective

Perfect wound healing includes not only integrity in appearance but also restoration of local tissue function. In the process of wound healing, bacteria as well as the reduction of inflammation, the regeneration of blood vessels, and the repair of re-epithelialization all play important roles. Through a systematic summary, The results show that NPs have bacteriostatic and anti-inflammatory activities (such as OLE, aloin, chamomile, aloe-emodin, etc.) and promote angiogenesis (such as AS-IV, asiaticoside, GA, apigenin, quercetin, etc.) and re-epithelialization (such as ginsenoside Rb1, lupeol, curcumin, ferulic acid, FVP, etc.). Selection of the corresponding pharmacological treatment, based on differences in the local characteristics of the wound, might be able to be more targeted.

In addition, due to the good ability of some drugs to promote wound healing, they have been widely used in the clinic, such as Centella Asiatica, Curcumin, Resina Draconis, and Quercetin. Clinical studies have shown that Centella Asiatica extract can promote skin wound healing after laser, improve skin texture and reduce pigmentation (Damkerngsuntorn et al., 2020), Besides, Curcumin has been widely used in the clinic. Such as capsules, nanofibers, transdermal patches, etc. Clinical studies have shown that compared with oral curcumin, local use seems to have a more obvious effect on wound healing [68 (Vollono et al., 2019), As a safe and easily available drug, dragon’s blood is an ideal choice for wound healing. Clinical studies have shown that the phenolic compounds in Longxue cream can shorten the inflammatory process and form a physical barrier to prevent microbial infection (Namjoyan et al., 2015). To improve diabetes foot ulcers, after using nano hydrogel embedded with quercetin and oleic acid, the wound healed completely within 1 month without adverse drug reactions. This indicates that the preparation can be used for the management of wound healing (Kant et al., 2021). The clinical effects of the above four natural products on wound healing have been verified, and further research and development of new clinical drugs will be the direction of efforts.

As research continues to deepen, certain natural products may be able to work together with matrices such as hydrogels, dressings, seeded scaffolds, fibrous mats, and so on. Such as AS-IV (Peng et al., 2012), FVP (Chen et al., 2021), APS (Yang et al., 2015), OLE (Samancio et al., 2016), BSP (Luo et al., 2010), Chamomile extract (Salehi et al., 2017). These novel products hold great promise in improving wound healing and are highly worth entering clinical research.

In general, Ginsenosides Rb1 (Zhang et al., 2021), Lupeol (Pereira Beserra et al., 2020), garlic acid (Singh et al., 2019), Ferulic acid (Dwivedi et al., 2022), Hesperdin (Jagetia and Rao, 2022), Aloin (Liu et al., 2015), Simmondsia cheinensis (Ranzato et al., 2011), Snail mucins (Gugliandolo et al., 2021), Angelica Sinensis ethanol extract (Bai et al., 2012b), Apigenin (Choi et al., 2016)have important potential to promote wound healing, and the molecular mechanism should be further studied. This conclusion is supported by desirable results, both in animal and cell experiments. Regrettably, both the mechanisms and targets through which the above natural products act warrant further exploration.

Taking a step further, besides focusing on natural product active ingredients, toxic side effects of drugs should also be paid attention to. Such as Aloe Emodin is phototoxic, hepatotoxic, and nephrotoxic (Dong et al., 2020). Toxicity also occurs when GL-PS exceeds the stimulatory concentration (Bishop et al., 2015). Honey extract is also partially toxic (Islam et al., 2014). Comfrey Extract has hepatotoxic and carcinogenic properties (Mei et al., 2010). How to maximize the extraction of active ingredients of drugs instead of toxicity is also a question that should be addressed in natural product extraction.

The presently developed derived products such as hydrogels, nanoparticles, scaffolds, and dressings have a promising future (Sun et al., 2018; Dart et al., 2019; Moeini et al., 2020; Sharma et al., 2020; Sharma et al., 2022)–(Sun et al., 2018; Dart et al., 2019; Moeini et al., 2020; Sharma et al., 2020; Sharma et al., 2022). Although the active ingredients of these natural medicines can effectively treat unhealed wounds, their effects depend on the advanced nature and suitable dosage of sophisticated technologies and instruments. Although several experiments have confirmed the significance of the therapeutic effect, it still needs to be further implemented in clinical practice. Regrettably, only the active ingredients of a single natural product are discussed, and whether the above drugs can be used in combination still deserves continued exploration. Cosmetically, increased scar proliferation, hyperpigmentation, and keratinization of the wound after healing also need to be taken into account.

## Data Availability

The original contributions presented in the study are included in the article/Supplementary Material, further inquiries can be directed to the corresponding authors.

## Abbreviations

AS, asiaticoside; ASEE, angelica sinensis ethanol extract; AS-IV, astragaloside IV; APS, astragalus polysaccharides; BSP, bletilla striata polysaccharide; col1, type I collagen; col3, type III collagen; COX, cyclooxygenase; ECM, extracellular matrix; EGF, epidermal growth factor; EGFR, epidermal growth factor receptor; FGF, fibroblast growth factor; FVP, flammulina velutipes; GA, gallic acid; GL-PS, ganoderma lucidum polysaccharide; IL-1, interleukin-1; LOX, lipoxygenase; NF-κB, nuclear factor; NPS, natural products; OLE, oleuropein; PDGF, platelet-derived growth factor; PPAR, peroxisome proliferator-activated receptor; RD, resina draconis; SOD, superoxide dismutase; SSF, snail secretory filtrate; TNF, tumor growth factor; UV, ultraviolet; VEGF, endothelial growth factor.
