# Supplementary material for: The potential application of natural products in cutaneous wound healing: A review of preclinical evidence
Source: Front Pharmacol. 2022 Jul 22;13:900439. doi: 10.3389/fphar.2022.900439 (PMC9354992; doi:10.3389/fphar.2022.900439)
Supplement: Supplementary file 1 [file Table1.pdf]

Flavonoids	NPs name	In vivo/vitro	Models	Range of dosage	Results	Reference
Saponin compounds	Ginsenoside Rb1	In vivo	Male adult Sprague–Dawley rats,second-degree burn injury model	1.25, 2.5, or 5 g/kg	↑PDGF-BB,PDGFR- β, FGF-2	(Zhang et al., 2021)
		In vivo	Male Balb/c mice,second-degree burn injury model	1 pg g^{-1}, 10 fg g^{-1} and 100 ag g^{-1} ointment	↑VEGF ,IL-1β,Increased macrophages	(Kimura et al., 2006)
		In vitro	HaCaT cells	100 fg ml−1, 10 pg ml−1 or 1 ng ml−1)	↑VEGF,HIF-1*α*,IL-1*β*	
	Astragaloside iv	In vivo	Sprague–Dawley male rats,The rat skin excision model	-	↓CollagenI/III	(Chen et al., 2012)
		In vitro	Keratinocytes	50 mol/L and 100 mol/L	↓TGF- 1	
		In vivo	Sprague-Dawley rats	4 mg / kg	Lesional skin lesions have reduced diameter, richer vascular network at the wound edge and less inflammatory infiltration.	(Shen et al., 2021)
		In vitro	Human umbilical vein endothelial cells (HUVECs)	6μmol / L	↑SUMO1,PCNA， Ras， HIF-1α，PPARγ,VEGFR2,HUVECs	

		In vivo	The rat full-skin excision model	pH 7.2±0.2<br>Viscosity 6.5±0.3Pa·s<br>Water absorptivity137±2.5%	↑TGF- β1	(Peng et al., 2012)
		In vivo	The rat full-skin excision model	20-30mg	↑Angiogenetic	(Chen et al., 2013)
	Asiaticoside	In vitro	Human dermal fibroblast cells	10Um,10,30,45min	Induced binding of smad2 and smad3	(Lee et al., 2006)
		In vivo	Guinea pigs<br><br>Sprague Dawley male rats,punch wound models<br><br>punch wounds,	0.05%, 0.1%, 0.2% twice daily for 7 days.<br><br>0.2 and 0.4%<br><br>20, 40<br>or 80 m g<br>compound(Methylcellulose disks)	56%increase in hydroxyproline, 57% increase in tensile strength, increased collagen content and better epithelisation.	(Shukla et al., 1999b)

		In vitro	Chick chorioallantoic membrane (CAM) model.	20,40,or 80 mg/disk	promoted angiogenesis	
		In vivo	The excision-type cutaneous wounds in rats	0.2%, topical	enhanced induction of antioxidant levels.	(Shukla et al., 1999a)
	Lupeol	In vivo	Normal keratinocytes and dermal fibroblasts	1-5μg/mL	↑ IL-6、TNFα、IL-8 , RANTES, Rho-GTP,p38-MAPK	(Wardecki et al., 2016)
		In vitro	The excision and incision wound models.	0.2% w/v gel(external), 1% w/v(Oral)	Increased indicating increase of collagenation and absence of monocytes.	(Harish et al., 2008)
		In vitro	Human neonatal foreskin keratinocytes and fibroblasts.	0.1, 1, 10, and 20 μg/mL	↓NF-Kb, ↑Akt，p38,Tie-2	[58]
		In vitro	The hyperglycemic rats	0.2%w / w	↓NF-κB, IL-6, Nf-κb, Vegf-A<br>↑IL-10, FGF-2，TGF-β1 ,collage-III, Hif-1α，Sod-2, Ho-1	(Lupeol, a Dietary Triterp

						ene, Enhances Wound Healing in Streptozotocin-Induced Hyperglycemic Rats with Modulatory Effects on Inflammation, Oxidative Stress, and Angiog

						enesis)
		In vitro	The excisional wounds in rats	0.1%, 0.2%,0.4%	↓TNF-a, IL-1β,IL-6）,NF-κB<br>↑VEGF, EGF, TGF-β1	(Pereira Beserra et al., 2020)
Polyphenolic Compounds	Curcumin	In vitro	Human gingival fibroblasts (hGFs).	0, 0.1, 1, 10, or 20 μm curcumin	↑KGF-1, ,EGFR, col1,	(Rujirachotiwat and Suttamanatwong, 2021)
		In vitro	linear incision and full-thickness excision wound models	1 mg	higher collagen content,<br>better granulation, higher wound maturity, dramatic decrease in superoxide dismutase, and slight in crease in catalase.	(Gong et al., 2013)
	Oleuropein	In vivo	Twenty four male Babl/c	a single daily dose of 50 mg/kg Oleuropein dissolved in distilled water for a total period of 7 days.	↑VEGF	(Mehraein et al., 2014)
		In vivo	Healthy adult male Spraque Dawley rats	20% Oleuropein,	Reduced wound healing time	(Samancio et

						al.)
	Gallic acid	In vivo	Excision and dead space wound models	5–2000 mg/Kg b.w.	Decreased the size of scar area and days of re-epithelialization	(Singh et al., 2019)
		In vitro	mouse embryonic fibroblasts.	GA（10，20，50，100 和 200μM	Accelerates the cell migration of keratinocytes and fibroblasts<br><br>↑FAK,JNK	(Gallic Acid Promotes Wound Healing in Normal and Hyperglucidic Conditions)
	Ferulic acid	In vivo	Incision and dead space wound healing models in rats	1% (w/w external),0.2mg/kg(oral)	Epithelisaion period were decreased	(Dwivedi et al.)
		In vivo	streptozotocin induced diabetic rats using excision wound model.	10 and 20 mg/kg(oral); 2%(external	The hydroxyproline And hexosamine content increased significantly	(Ghaisas et al., 2014)

Flavonoids	Apigenin	In vivo	skin damage model of the female BALB/c mice	0.1g	↓TNF-α，IL-1β,IFN-γ	(Cheng et al., 2018)
			Flap model	0.5 mg/mL	improving survival rate of random skin flaps	(Zhu et al., 2021)
	Quercetin	In vivo	Pelargonium dorsal skin excision model	10-100uM	↓Co,Lo	(Kim et al., 1998)
		In vivo	Diabetic rats	0.3%	↓TNF-α，IL-1β, MMP-9<br>↑IL-10, VEGF, TGF-β_1<br>TNF-α，IL-1β	(Topical application of quercetin improves wound repair and

						regeneration in diabetic rats: Immunopharmacology and Immunotoxicology: Vol 43, No 5, 5)
	Hesperidin	In vivo	Diabetic Sprague Dawley rat model (male, 180-220 g)	25, 50 and 100 mg/kg, p.o	↑VEGF-c, Ang-1 / Tie-2, TGF-β, Smad-2 / 3 mRNA	(Li et al., 2018)
		In vivo	full thickness circular excision wound	1%-10%	↓NF-κB、COX-II, LOX	(Jagetia and Rao, 2017)

		In vivo	Old female hairless mice	60 µl of 2% hesperidin or 70% ethanol	Enhances epidermal permeability barrier	(Hou et al., 2012)
Anthraquinone compounds	Aloin	In vitro	Murine macrophage RAW264.7 Cells	10, 20, 50, 75, 100, 150 and 200 µg/ml	↓JAK1-STAT1/3 signaling pathway	(Ma et al., 2018, 7)
		In vitro	Human skin fibroblast Hs68 cells.	150 or 300 M	↓ROS , 8-OH-dG	(Liu et al., 2015)
		In vitro	HaCaT cells<br>Hairless mice model	1,5µM and10µM<br>0.1% and 0.5%	↑IL-1β, IL-6, TGF-β1, TNF-α	(Wahedi et al., 2017)
	Aloe Emodin	In vivo	Rat burn wound model	1, 100 and 500 ng/ml	↑VEGF, IL-1β ,MCP-1,	(Lin et al., 2016)
Polysaccharides	Bletilla striata polysaccharide		The murine macrophage cell line RAW264.7	5-500 µg/ml	CD31, α-SMA, and CK7	(Bletilla striata Polysaccharide

						Stimulates Inducible Nitric Oxide Synthase and Proinflammatory Cytokine Expression in Macrophages - Science Direct)
		In vivo	Full-thickness trauma mouse model	The 12.5%-crosslinked BSP hydrogel with a thickness of 0.5 mm was introduced onto the wound bed.	↑EGF,↓ TNF-α	(Luo et al., 2010)
		In vitro	Primary HUVECs	0.5 mg/mL BSP or 1 mg/mL BSP	↑VEGF	

	Flammulina velutipes	In vitro	The murine macrophage cell line RAW264.7	5,25,50,100,200,500ug/ml	IL-1β, IFN-γ, TNF-α	(Xu et al., 2019)
		In vivo	The rat full-thickness skin wound model,	600 μm thickness and 15 mm diameters	↑CD31、α-SMA,CK7	(Chen et al., 2021)
	Ganoderma lucidum polysaccharides	In vivo	The full thickness excisional wound	10, 50 or 250 mg/kg/day	↓p66Shc, MnSOD	(Tie et al., 2012)
		In vitro	Primary human skin fibroblasts	0, 10, 20, 40, 80, and 160 μg/mL of GL-PS	Wnt/β-catenin signaling pathway, ↑TGF-β1	(Hu et al., 2019)
	Astragalus polysaccharide	In vitro	Human skin fibroblast cells (CCC-HSF-1; HSF), The scalded mice model	0，1，5 and 25mg / l	↓Cyclin D1, IκBα, ↑TGF-β1,BFGF,EGF	(Zhao et al., 2017)
		In vivo	The diabetic rat model	5 mg	↑SBF, VECs	(Yang et al., 2015)

Others	Honey extract	In vivo	The full-thickness burn wound in rats	200 µl	↓TNF-α, NF-κB, IL-1β, COX-2, substance-P	(Yadav et al., 2018)
		In vivo	rat aortic ring assay	5-0.008%v/v	Promote angiogenesis	(Rossiter et al., 2010)
	Comfrey Extract	Clinical research	278 patients with fresh abrasions	10 % Extrat	The wound healing speed is accelerated and the area is reduced.	(Staiger, 2012)
		Clinical research	108 children aged 3–12 years	1%-10%	The wound area was reduced	(Barna et al., 2012)
	Chamomile extract	In vitro	Murine RAW 264.7 macrophages	0-40ug/mL	PGE2, COX-1 and COX-2, NF-κB	(Srivastava et al., 2009)
		In vivo	adult male Wistar rats	100g crashed flower and 100ml olive oil	The wound area decreases and the tissue regeneration capacity increases	(Jarrahi, 2008)
		In vivo	Rats	0.04ml/Day	Wound reepithelialization, collagen fibrillogenesis	(Duarte et al., 2011)
		In vivo	A rat third-degree burn wound	hydrogel/extract/4 wt% nZ	Decreased wound size, collagen	(Salehi

			model	(hydrogel NZE)	formation, angiogenesis,	et al., 2017)
		In vitro	fibroblast mouse cells (L929)	starch/4 wt% nZ and starch/extract/4 wt% nZ	Cells are more mature	
	Resina Draconis	In vivo	Excision Wound Model	5%	Increased the fibroblast growth, collagen synthesis, and the healing process.	(Liu et al., 2013)
		Clinical research	The patients, aged between 14 years and 65 years	15%	Shorten wound healing time.	(Namjoyan et al., 2015)
	Simmondsia chinensis	In vitro	HaCaT keratinocytes and human dermal fibroblasts	0.0-5%JWL	↑PI3K-Akt-mTOR pathway, p38 and ERK1/2 MAPKs.	(Ranzato et al., 2011)
	snail mucins	In vivo	CD1 mice (male, 20–30 g); Full-Thickness Excisional Wound Model	400μLSF	↓MMP-1， MMP-2,MMP-9, MPO, IL-1β,TNF-α<br><br>↑COL3A1<br>VEGF,α-sma	(Gugliandolo et al., 2021)
	Angelica Sinensis ethanol extract	Clinic research	human normal fibroblasts (Fb)	5 × 10(-4) to 5 × 10(-2) g/L	↑collagen I, collagen III	(Bai et al., 2012a)
